# Chromatin Accessibility Landscape of Human Triple-negative Breast Cancer Cell Lines Reveals Variation by Patient Donor Ancestry

**DOI:** 10.1158/2767-9764.CRC-23-0236

**Published:** 2023-10-05

**Authors:** Alexandra R. Harris, Gatikrushna Panigrahi, Huaitian Liu, Vishal N. Koparde, Maeve Bailey-Whyte, Tiffany H. Dorsey, Clayton C. Yates, Stefan Ambs

**Affiliations:** 1 Laboratory of Human Carcinogenesis, Center for Cancer Research, NCI, NIH, Bethesda, Maryland.; 2 Center for Cancer Research Collaborative Bioinformatics Resource Frederick National Laboratory for Cancer Research, NCI, Frederick, Maryland.; 3 Advanced Biomedical Computational Sciences, Frederick National Laboratory for Cancer Research, Leidos Biomedical Research, Inc., Frederick, Maryland.; 4 School of Medicine, University of Limerick, Limerick, Ireland.; 5 Department of Pathology, Johns Hopkins University School of Medicine, Baltimore, Maryland.

## Abstract

African American (AA) women have an excessive risk of developing triple-negative breast cancer (TNBC). We employed Assay for Transposase-Accessible Chromatin using sequencing to characterize differences in chromatin accessibility between nine commonly used TNBC cell lines derived from patients of European and African ancestry. Principal component and chromosome mapping analyses of accessibility peaks with the most variance revealed separation of chromatin profiles by patient group. Motif enrichment and footprinting analyses of disparate open chromatin regions revealed differences in transcription factor activity, identifying 79 with ancestry-associated binding patterns (FDR < 0.01). AA TNBC cell lines exhibited increased accessibility for 62 transcription factors associated with epithelial-to-mesenchymal transition, cancer stemness/chemotherapeutic resistance, proliferation, and aberrant p53 regulation, as well as KAISO, which has been previously linked to aggressive tumor characteristics in AA patients with cancer. Differential Assay for Transposase-Accessible Chromatin signal analysis identified 1,596 genes located within promoters of differentially open chromatin regions in AA-derived TNBC, identifying DNA methyltransferase 1 as the top upregulated gene associated with African ancestry. Pathway analyses with these genes revealed enrichment in several pathways, including hypoxia. Culturing cells under hypoxia showed ancestry-specific stress responses that led to the identification of a core set of AA-associated transcription factors, which included members of the Kruppel-like factor and Sp subfamilies, as well as KAISO, and identified *ZDHHC1*, a gene previously implicated in immunity and STING activation, as the top upregulated AA-specific gene under hypoxia. Together, these data reveal a differential chromatin landscape in TNBC associated with donor ancestry. The open chromatin structure of AA TNBC may contribute to a more lethal disease.

**Significance:**

We identify an ancestry-associated open chromatin landscape and related transcription factors that may contribute to aggressive TNBC in AA women. Furthermore, this study advocates for the inclusion of diversely sourced cell lines in experimental *in vitro* studies to advance health equity at all levels of scientific research.

## Introduction

Human cancer cell lines are valuable and widely used *in vitro* model systems essential to performing cancer research at its most reductionist level. Under the proper growth conditions, cancer cell lines can retain many of the genetic properties of the parental tumor from which they were derived (1). Representing a seemingly inexhaustible source of biologic material, these cost-effective and manipulatable systems remain essential to mechanistic studies, drug discovery efforts, and preclinical research. Because of the foundational role they play, researchers now recognize that issues occurring at this most basic level can result in ripple effects throughout the subsequent scientific pipeline, undercutting data reproducibility, accuracy, and quality. A recent example by Hooker and colleagues tested genetic ancestry of 15 common cell lines, uncovering several misclassification errors in ancestry that may contribute to erroneous conclusions in the health disparities field (2). Ultimately, inaccuracies and missteps at this earliest level could lead to spurious drug candidates that progress to preclinical development only to fail, squandering resources and time.

Despite the centrality of cancer cell lines to biomedical research and development, these *in vitro* systems have been historically sourced from the tumors of predominantly White/European American (EA) patients (3). Indeed, the lack of racial and ethnic diversity and representation in cell lines origin could impede discovery efforts that benefit all populations, impacting health equity even at this most basic stage. Yet, little has been done to directly test whether donor ancestry does in fact impact the molecular properties of cancer cell lines in culture at baseline or after experimental perturbation.

In this study, we sought to characterize differences in chromatin accessibility in triple-negative breast cancer (TNBC) cell lines derived from women of two different ancestral backgrounds—EA and African American (AA). We chose to focus our investigation on TNBC due to the well-documented racial disparities in tumor biology and breast cancer survival (4). AA women are at increased risk of developing and dying from TNBC, an aggressive breast cancer subtype, compared with EA women in the United States. Further investigation into biological factors that contribute to these disparities is needed to fully understand this multifactorial problem, including more focus on the epigenetics of racial/population diversity and its influence on breast cancer biology and outcomes. Some studies seeking to investigate ancestry as a biological contributor to breast cancer disparities have suggested that the extent of African ancestry itself correlates with the likelihood of being diagnosed with more aggressive breast cancer subtypes than women from other population groups (5). Yet, many U.S.-based studies rely on self-reported race, a social construct, as a pseudoproxy for genetic ancestry, which is intrinsically biological (6). Recent work by Martini and colleagues has begun to disentangle the two, finding 50% of gene expression associated with ancestry was distinct from that of self-reported race (7). Here, we adopt a reductionist approach that employs widely used TNBC cell lines to explore the impact of differential ancestry-associated chromatin profiles on transcription factor (TF) activity, downstream gene expression, and their implications for tumor biology. Knowledge gained here could hold promise to advance our understanding of biological contributors to cancer disparities and support the importance of diversity and inclusion within *in vitro* systems.

## Materials and Methods

### Cell Lines and Sample Preparation Methods

#### Cell Lines and Culture

All human breast cancer lines were obtained from ATCC. MDA-MB-157 (ATCC catalog no.: HTB-24; RRID: CVCL_0618), MDA-MB-231 (ATCC catalog no.: HTB-26; RRID: CVCL_0062), MDA-MB-436 (ATCC catalog no.: HTB-130; RRID: CVCL_0623), and HCC1806 (ATCC catalog no.: CRL-2335; RRID: CVCL_1258) cells were grown in DMEM (Gibco Laboratory) with 2 mmol/L glutamine (Millipore Sigma) and 10% FBS (HyClone Laboratories). MDA-MB-468 (ATCC catalog no.: HTB-132; RRID: CVCL_0419), Hs578T (ATCC catalog no.: HTB-126; RRID: CVCL_0332), HCC70 (ATCC catalog no.: CRL-2315; RRID: CVCL_1270), BT549 (ATCC catalog no.: HTB-122; RRID: CVCL_1092), and HCC2157 (ATCC catalog no.: CRL-2340; RRID: CVCL_1261) cells were grown in RPMI media (Gibco Laboratory) supplemented with 10% FBS. HCC2157 cells grow in suspension whereas all other cell lines are adherent. We obtained authentication of these cell lines through ATCC services using a short tandem repeat analysis; as cell lines were obtained directly from ATCC, *Mycoplasma* testing was not performed. The biological sex of all cell lines was female. To perform the Assay for Transposase-Accessible Chromatin using sequencing (ATAC-seq) analysis, following regular cell culture procedure, one million cells (MDA-MB-157, MDA-MB-231, MDA-MB-436, HCC1806, MDA-MB-468, Hs578T, HCC70, BT549, and HCC2157) were suspended in 1.5 mL freezing medium (basal media + 20% FBS + 10% DMSO). The above cryopreserved cells were sent to the Sequencing Facility at Frederick National Laboratory for Cancer Research, Frederick, MD, where sample processing and sequencing was done.

#### Cell Culture Under Hypoxia

For normoxic (21% O_2_) conditions, cells (MDA-MB-157, MDA-MB-231, HCC1806, MDA-MB-436, MDA-MB-468, Hs578T, HCC70, BT549) were cultured at 37°C in a 5% CO_2_ humidified environment as an adherent monolayer. Hypoxia experiments were performed with the above eight adherent cell lines in a hypoxia chamber (BioSpherix) at 1% O_2_ at 37°C in a 5% CO_2_-humidified environment. Briefly, breast cancer cells were plated in T75 flask (1 × 10^6^ cells/flask). Media was changed after 24 hours, and cells were exposed to normoxia (21% O_2_) and hypoxia (1% O_2_) for 48 hours. Experiments were conducted in duplicates. After 48 hours, the cells were harvested from the flasks by 0.25% trypsin (Gibco Laboratory) and 1 million cells were suspended in 1.5 mL of freezing medium (basal media + 20% FBS + 10% DMSO). The above cryopreserved cells were sent to the Sequencing Facility at Frederick National Laboratory for Cancer Research, Frederick, MD, where sample processing and sequencing were done.

#### Nuclei Isolation and ATAC-seq

Cell revival, cell lysis, transposition, and DNA extraction were performed using a published method by Corces and colleagues, 2017 (8) with some modifications for cryopreserved cell processing (9). Briefly, frozen cell pellets of 50,000 cells were resuspended in 1 mL of cold ATAC-seq resuspension buffer (RSB; 10 mmol/L Tris-HCl pH 7.4, 10 mmol/L NaCl, and 3 mmol/L MgCl_2_ in water). Cells were centrifuged at 500 rcf for 5 minutes in a prechilled (4°C) fixed-angle centrifuge. After centrifugation, 900 µL of supernatant was aspirated, which left 100 µL of supernatant. This remaining 100 µL of supernatant was carefully aspirated by pipetting with a P200 pipette tip to avoid the cell pellet. Cell pellets were then resuspended in 50 µL of ATAC-seq RSB containing 0.1% NP40, 0.1% Tween-20, and 0.01% digitonin by pipetting up and down three times. This cell lysis reaction was incubated on ice for 3 minutes. After lysis, 1 mL of ATAC-seq RSB containing 0.1% Tween-20 (without NP40 or digitonin) was added, and the tubes were inverted to mix. Nuclei were then centrifuged for 10 minutes at 500 rcf in a prechilled (4°C) fixed-angle centrifuge. Supernatant was removed with two pipetting steps, as described before, and nuclei were resuspended in 50 µL of transposition mix by pipetting up and down six times. Transposition reactions were incubated at 37°C for 30 minutes in a thermomixer with shaking at 1,000 rpm. Reactions were cleaned up with Zymo DNA Clean and Concentrator 5 columns. The remainder of the ATAC-seq library preparation was performed as described previously (9). All libraries were amplified with a target concentration of 20 µL at 4 nmol/L, which is equivalent to 80 femtomoles of product. A total of 32 ATAC-seq samples were pooled and sequenced on NextSeq using OMNI ATAC-seq protocol adapted from Corces and colleagues (8) and paired-end sequencing. All samples have >95% Q30 bases, which is defined as the percentage of bases called with an inferred accuracy of 99.9% or above and a measure of base calling quality. All the samples had yields between 48 and 417 million pass filter reads. Samples were trimmed for adapters using Cutadapt v.4.4 (ref. 10; RRID: SCR_011841) before the alignment. The trimmed reads were aligned with the human genome assembly – hg38 reference using Bowtie2 v.2.5.1 (11) alignment (RRID: SCR_016368). Overall alignment percentage for the samples was 97% and unique alignment was above 53%. There were 0.96% to 8.08% unmapped reads. Library complexity was measured by uniquely aligned reads using Picard's MarkDuplicate utility. All the samples had library complexity with percent nonduplicated reads ranging from 38% to 69%. The peaks were called using Genrich and total number of peaks ranged between 54,835 and 83,046. The number of reads falling in peaks (FRiP score) generally ranged between 30% and 70%. The ATAC-seq data for the human breast cancer cell lines were deposited in the NCBI Gene Expression Omnibus (GEO) database under accession number GSE223182.

#### RNA Extraction for Gene Expression Studies

RNA was isolated using TRIzol and subsequently DNase treated. Integrity of isolated RNA was evaluated using RNA ScreenTapeon the Agilent Tapestation (Agilent Technologies). Sequencing was performed on an Illumina NextSeq 2000 machine at the Sequencing Facility, Leidos Biomedical Research, Inc., Frederick National Laboratory for Cancer Research using standard protocols. Briefly, 16 mRNA sequencing samples were pooled and sequenced on NextSeq 2000 P2 using Illumina Stranded mRNA Ligation Kit and paired-end sequencing. The samples had 56 to 69 million pass filter reads with more than 95% of bases above the quality score of Q30. The RNA sequencing (RNA-seq) data for the human breast cancer cell lines were deposited in the NCBI's GEO database under accession number GSE223181.

### Chromatin Accessibility Analysis

#### ATAC-seq Data Processing

After verifying that the pair-end reads are of acceptable sequencing quality, sequencing adapters and primers were trimmed away using Cutadapt (10). Trimmed reads that align to known blacklisted regions using Bowtie2 (11) were excluded from downstream analysis. Bowtie2 was again applied to align the remaining reads to the host genome allowing “multimappers”. These latter sequences were then mapped as recommended by ENCODE guidelines (12). Reads which remained unmapped or failed quality checks or were identified as a PCR duplicate are removed. Primary alignments of the remaining reads were further filtered to remove low MAPQ alignment. Reads were then deduplicated and parsed to Genrich (13) for ATAC-seq peak calling. FastQC v.1.0.0 (ref. 14; RRID: SCR_014583) for base calling statistics, PRESEQ (15) for library complexity, and fragment length distribution, transcription start site enrichment, fraction of reads in peaks, nucleosome-free-region to mononucleosome-region read ratio and other widely recommended (16) ATAC-seq–specific quality control metrics were applied to verify acceptable quality of all sample replicates. Peaks called by Genrich (13) for each cell line were converted to fixed-width peaksets and peaks reproducible across replicates are labeled as consensus peak calls. Consensus peaks from all cell lines were renormalized and merged to generate a preliminary list of regions of interest (ROI; ref. 17). Using the prefiltered alignment files from above, featureCounts v.2.0.6 (18) was used to create a count matrix counting the Tn5-nicking sites (19) in these ROIs. ChIPSeeker v.1.36.0 (ref. 20; RRID: SCR_021322) was used to annotate the ROIs in relation to known gene body loci.

#### Principal Component Analysis

The dimensionality of the Tn5-nicking site counts matrix was reduced by calculating the principal components and plotting the first two components which explain a significant proportion of the overall variation.

#### Identification of Differentially Accessible Regions

Loading the count matrix into DESeq2 v.1.45.5 (ref. 21; RRID:SCR_015687) a differential analysis was then performed to determine the ROIs which show significant differences in measured ATAC-seq signal between EA and AA samples. The results were filtered for differential regions with FDR ≤ 0.05 and signal fold change ≥ 2. This allowed us to filter the overall open chromatin regions of interest into those with significant differential ATAC signals between the two groups or differentially accessible regions (DAR).

#### TF Binding Motif Enrichment

We used ChromVAR v.1.22.1 (22) to estimate bias-corrected deviations of TF binding motif enrichment. It enables accurate clustering of ATAC-seq profiles and characterization of motifs associated with variation in chromatin accessibility. The motifmatchr package was used to match motifs from the JASPAR core database (23) to peaks from differentially open chromatin regions and variability (SD of the z-scores computed across all samples for a set of peaks) and adjusted *P* values were calculated, and the top 50 most variable TFs were plotted on a heat map.

#### Digital Footprinting Analysis

Using the motifs from the HOCOMOCO (24) database (RRID: SCR_005409), we ran TOBIAS v.0.14.0 (25) in the identified DARs to predict TF occupancy or perform digital footprinting analysis. TOBIAS results shed light on gain and loss of individual TF footprints in the DARs. TFs with a differential binding score above |0.225| and a FDR < 0.01 were considered significant.

#### Pathway Enrichment Analyses

For pathway enrichment analysis, ancestry-related genes upregulated in either EA or AA TNBC, which were identified through differential analysis of ATAC signal by DESeq2 (|log_2_FC| > 2, FDR < 0.01), were then imported into the Enrichr tool (ref. 26; RRID: SCR_001575) to perform an overrepresentation analysis of both cancer hallmark pathways (MSigDb, Broad Institute; RRID: SCR_016863) and Kyoto Encyclopedia of Genes and Genomes (KEGG) pathways (RRID: SCR_012773). Pathways with an FDR < 0.2 were used for subsequent biologic interpretation.

#### STRING Protein–protein Interaction Network Construction of Ancestry-specific Transcriptional Factors

Hypoxia-induced and ancestry-specific differentially bound TFs identified by TOBIAS were analyzed using the STRING database (RRID: SCR_005223) to identify groups of TF proteins that have known and/or predicted relationships (27). Using STRING, we performed pathway enrichment analysis, which identified overrepresentation of Reactome (RRID: SCR_003485) pathways of functional subsystems that are observed more frequently than expected using hypergeometric testing against a statistical background of the genome (27). Network nodes signify proteins and colored edges denote evidence of protein–protein interactions (PPI) of various types. Networks were clustered via the Markov cluster algorithm with default inflation parameters.

### RNA-seq Data Processing and Analysis

The Illumina FASTQ files were assessed for quality control using FastQC v.0.11.6. Reads of the samples were trimmed for adapters and low-quality bases using Cutadapt before alignment with the reference genome (hg38) and the annotated transcripts using STAR v.2.7.5b. The gene expression quantification was performed using RSEM v1.3.2. The average mapping rate of all samples was 97% and unique alignment was above 88%. The mapping statistics were calculated using Picard software. Percent coding bases were between 51% and 61%. Percent untranslated region bases were 32%–43%, and mRNA bases were between 89% and 94% for all the samples. Library complexity was measured in terms of unique fragments in the mapped reads using Picard's MarkDuplicate utility. The samples had 58%–71% nonduplicate reads. Differential expression analysis was done using DESeq2 package in R. Differential expression genes (DEG) were filtered by a *q* value (FDR) < 0.05, the absolute value of fold change > 2. We then performed gene set enrichment analysis (GSEA) using the gene transcription regulation reference (Gene Transcription Regulation Database: GTRD) from the Molecular Signatures Database: http://www.gsea-msigdb.org/gsea/msigdb/human/collections.jsp).

### Ancestry Estimation for Cell Lines Using RNA-seq Data

Germline variants were called using GATK's HaplotypeCaller v.4.2.6.1 from available RNA-seq data for eight cell lines cultured under normoxic conditions, MDA-MB-468, MDA-MB-157, HCC1806, HCC70, MDA-MB-231, MDA-MB-436, Hs578T, and BT549. For admixture analysis, only SNPs that were biallelic were retained in the analysis. We used GRAF-pop v.1.0 (https://www.ncbi.nlm.nih.gov/projects/gap/cgi-bin/Software.cgi), which is a fast distance–based method to infer ancestry based on references from multiple genotype datasets, including those of populations of European, African, and Asian descent. The method assigned a predominant African ancestry to MDA-MB-468, MDA-MB-157, HCC1806, and HCC70 cells and a predominant European ancestry to the MDA-MB-231, MDA-MB-436, Hs578T, and BT549 cells, consistent with donor ethnicities reported by ATCC for these cell lines. Ancestry estimates of our cell lines aligned closely with publicly available data from the Estimated Cell Line Ancestry (ECLA) database from the Moffitt Cancer Center (http://ecla.moffitt.org).

### The Cancer Genome Atlas Validation

To validate ancestry-specific upstream transcriptional regulators identified by ATAC-seq in cell line analyses, RNA-seq data from The Cancer Genome Atlas (TCGA; RRID: SCR_003193) breast cancer data were downloaded from the Broad Institute's GDAC (https://gdac.broadinstitute.org/runs/stddata__2016_01_28/data/BRCA/20160128/) covering 112 TNBC patient tumors (*n* = 78 EA, *n* = 34 AA participants) to infer differences in TF activity by genetic ancestry, which was assigned using previously reported quantified genetic ancestry estimates (28). Of note, quantified genetic ancestry estimates were concordant with self-reported race in these subjects (Supplementary Fig. S1). Significant ancestry-specific genes upregulated in either EA or AA TNBC identified through differential analysis of TCGA BRCA TNBC RNA-seq (by DESeq2, |FC| > 1.5, FDR < 0.05) were input into the Ingenuity Pathways Analysis (RRID: SCR_008653) to perform an upstream regulator analysis.

### Statistical Analysis

Two experimental replicates of each cell line were submitted for ATAC- and RNA-seq and averaged for analyses. If not specified, R platform was used to compute statistics and generate plots. log_2_ fold change, *P* values, and FDR were calculated and used to assess significance. Significant differences for all quantitative data were considered when |log_2_FC| > 2 and FDR < 0.01 unless otherwise noted.

### Data Availability

The RNA-seq data for the human breast cancer cell lines were deposited in the NCBI GEO database under accession number GSE223181. The ATAC-seq data for the human breast cancer cell lines were deposited in the NCBI GEO database under accession number GSE223182.

GSE223183: This SuperSeries is composed of the following SubSeries:
GSE223181 Integrated chromatin accessibility and gene expression landscape of human TNBC cell lines reveals variation by patient donor ancestry [RNA-seq]
GSE223182 Integrated chromatin accessibility and gene expression landscape of human TNBC cell lines reveals variation by patient donor ancestry [Bulk ATAC-seq]

The following secure token has been created to allow review of record GSE223183 while it remains in private status: *urgfuuiiprstfkj*.

## Results

### Genome-wide Landscape of Chromatin Accessibility in EA- and AA-derived TNBC Cell Lines

**FIGURE 1:**
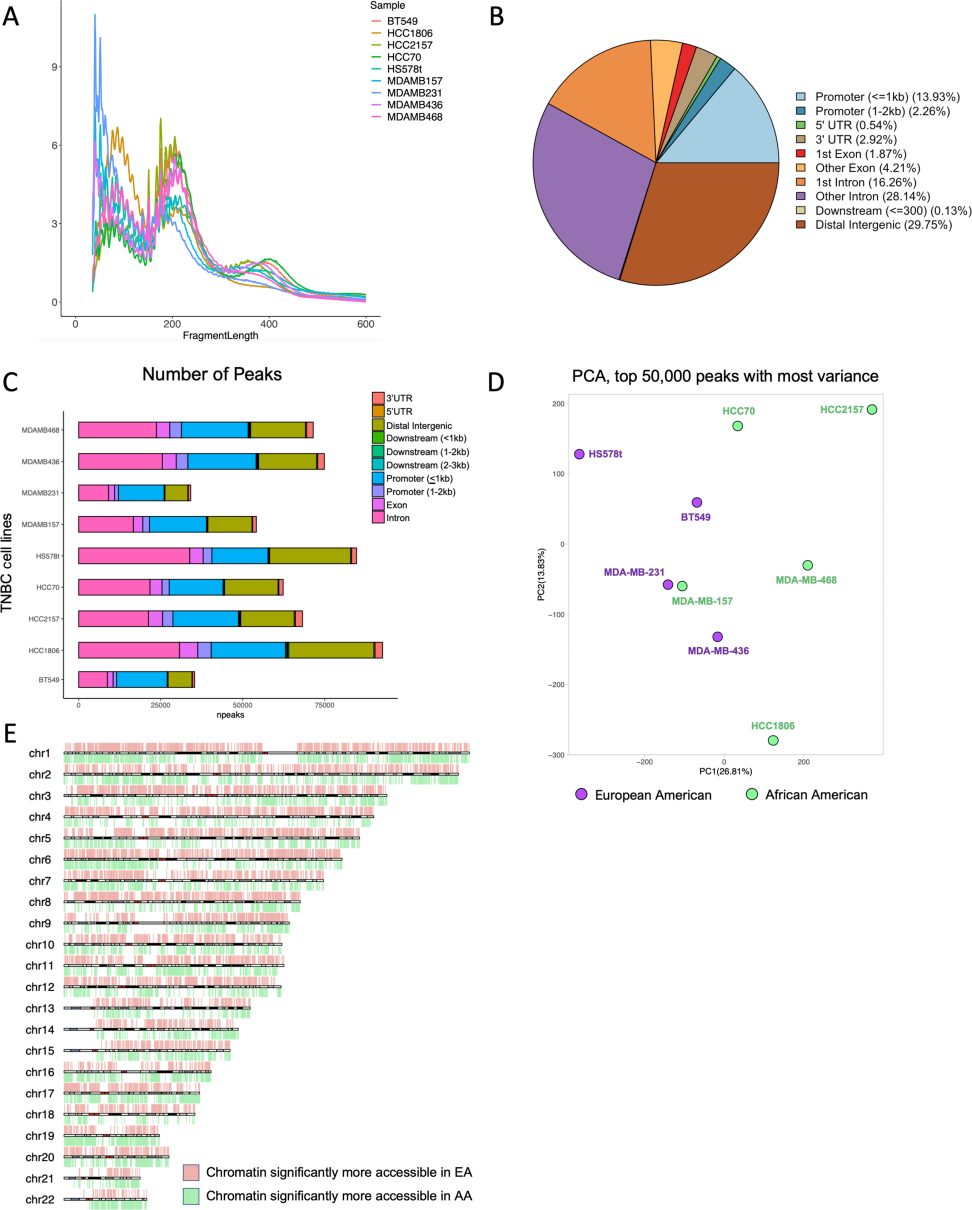
Genome-wide landscape of chromatin accessibility in TNBC cell lines derived from EA and AA women. **A,** Fragment length distribution plot for TNBC cell lines. Peak < 150 bp indicates a nucleosome-free peak; peak between 150 and 300 bp indicates a mononucleosome peak. FRiP scores greater than 0.3 indicate high quality of the data. Technical replicates (*n* = 2) were averaged for each cell line. **B,** Summary of peak distribution across all samples. **C,** Number of peaks by TNBC cell line. All samples had high peak numbers (∼75,000) covering promoters and intergenic regions. **D,** Principal component analysis showing separation of the chromosome accessibility landscape by ancestry for the nine TNBC cell lines. Technical replicates (*n* = 2) were averaged for each line. **E,** Chromosome map showing the distribution of DARs between EA and AA TNBC cells (FC > 2, FDR < 0.01).

The assay for transposase-accessible chromatin using sequencing, known as ATAC-seq, is currently the prevailing method used to assess chromatin accessibility across the genome (29). Genomic DNA is exposed to a highly active transposase, Tn5, which fragments DNA and inserts into open chromatin regions, adding sequencing primers. Upon sequencing, researchers can use the position of Tn5 to characterize open and closed chromatin sites, investigate TF binding, and infer downstream gene expression and regulation. In this study, we employed ATAC-seq to characterize differences in chromatin accessibility between EA (*n* = 4) and AA (*n* = 5) donors of nine commonly used TNBC cell lines (Table 1). Ancestry estimation (http://ecla.moffitt.org) indicates an average European ancestry of 93% for the four EA TNBC cell lines and 79.5% West African ancestry for the five AA TNBC cell lines (Table 1). Our ancestry estimates using the RNA-seq data from these cell lines were consistent with these reported estimates. ATAC libraries were generated using two replicates of each cell line. Chromatin was fragmented into the expected nucleosome-free regions and mononucleosome, dinucleosome, and trinucleosome patterns (Fig. 1A), with the fractions of reads in peaks (FRiP) falling between 30% and 70%, indicating high-quality data. The total number of peaks merged across nine cell lines was 212333, with intronic, intergenic, and promoter regions representing the most accessible genomic elements to Tn5 transposase (Fig. 1B and C). Our principal component analysis of the top 50,000 peaks with the most variance revealed separation of chromatin profiles by genetic ancestry (Fig. 1D), with EA TNBC showing a closer relationship to one another than AA TNBC. Genome-wide analysis of differentially accessible chromatin sites (FDR < 0.01 and |log_2_FC| > 2) in EA compared with AA TNBC cells confirmed distinctive patterning of open chromatin across a number of chromosomes (Fig. 1E). Together, these data suggest genome-wide differences in the chromatin landscape by donor ancestry in these commonly used TNBC cell lines.

**TABLE 1:** TNBC cell lines derived from women of EA and AA descent

Cell line	Donor ancestry	African^a^	European^a^	Asian^a^	ER	PR	HER2	TNBC subtype^b^	Morphology^b^	Tumor source^b^	Parental tumor histology^b^	Mutations^b^
BT549	European American	0.20%	89.00%	10.90%	Neg	Neg	Neg	Mesenchymal	Mesenchymal-like	Primary	Ductal carcinoma	PTEN RB1 TP53
MDA-MB-231	European American	2.00%	88.20%	9.80%	Neg	Neg	Neg	Mesenchymal stem-like	Mesenchymal-like	Metastasis, pleural effusion	Adenocarcinoma	BRAF CDKN2A KRAS NF2 TP53
MDA-MB-436	European American	1.40%	96.40%	2.20%	Neg	Neg	Neg	Mesenchymal stem-like	Mesenchymal-like	Metastasis, pleural effusion	Adenocarcinoma	BRCA1 RB1
HS578t	European American	0.50%	97.70%	1.70%	Neg	Neg	Neg	Mesenchymal	Mesenchymal-like	Primary	Carcinoma	CDKN2A HRAS PIK3R1 TP53
MDA-MB-157	African American	77.90%	17.40%	4.70%	Neg	Neg	Neg	Mesenchymal stem-like	Mesenchymal-like	Metastasis, pleural effusion	Medulallary carcinoma	NF1 TP53
MDA-MB-468	African American	80.30%	13.50%	6.20%	Neg	Neg	Neg	Basal-like 1	Basal-like	Metastasis, pleural effusion	Adenocarcinoma	PTEN RB1 SMAD4 TP53
HCC1806	African American	80.70%	15.50%	3.80%	Neg	Neg	Neg	Immunomodulatory	Basal-like	Primary	Primary acantyolytic squamous cell carcinoma	CDKN2A KDM6A STK11 TP53
HCC2157	African American	87.80%	7.80%	4.40%	Neg	Neg	Neg	Basal-like 1	Basal-like	Primary	Primary ductal carcinoma	KDM6A TP53
HCC70	African American	70.90%	23.70%	5.50%	Neg	Neg	Neg	Basal-like 2	Basal-like	Primary	Primary ductal carcinoma	PTEN TP53

^a^Ancestry estimates derived from the ECLA database from the Moffitt Cancer Center. Our ancestry estimates used our RNA-seq data from these cell lines and were concordant with ECLA.

^b^Cell line annotations obtained from the ATCC.

### EA- and AA-derived TNBC Cell Lines Exhibit Differential TF Accessibility by Donor Ancestry

**FIGURE 2:**
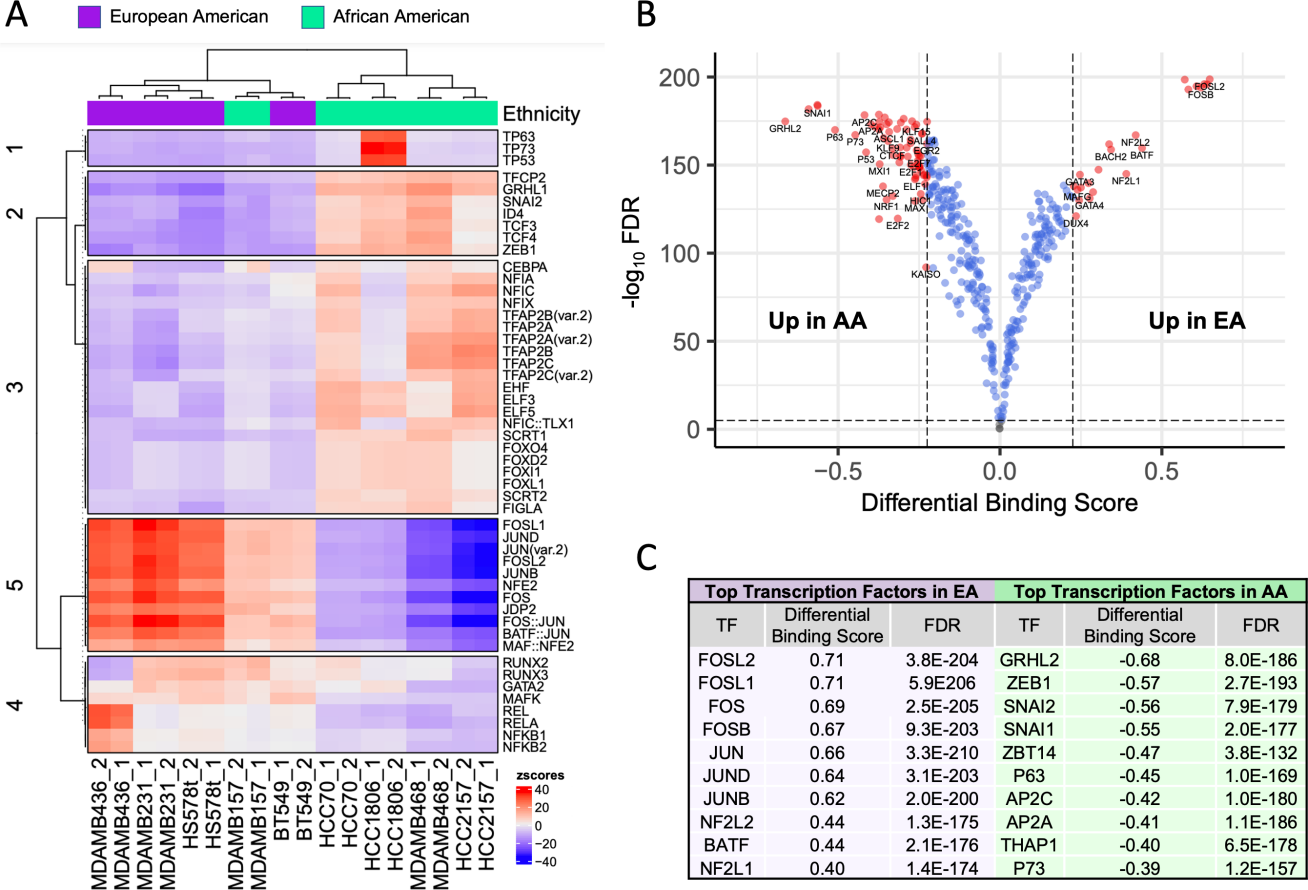
Patterns of differential TF binding in open chromatin regions among TNBC cell lines derived from EA and AA women. **A,** Overabundance of known TF motifs within differentially open regions. Heat map of top 50 most variable TFs by cell line donor ancestry based on differential ATAC signal analysis. **B,** Volcano plot of predicted differences in TF binding scores by patient group using digital footprinting analysis with TOBIAS. Open chromatin regions defined by TF binding sites are significantly different between cell lines from AA and EA patients. A total of 79 TFs were differentially bound in AA versus EA TNBC lines (points in red indicate TFs with differential binding score > |0.225| and FDR<0.01). A differential binding score of greater than 0.225 indicates increased binding in EA. A differential binding score of less than −0.225 indicates increased binding in AA. **C,** Top-ranked 10 TFs with differential binding capacities in the two donor groups identified through digital footprinting analysis.

TF dysregulation can induce aberrant gene expression associated with cancer; indeed, TF activity is altered in a number of cancers and has long been considered a lofty yet challenging candidate for drug targeting (30). Given their critical role in tumor initiation and progression, we focused our investigation on understanding differences in TF abundance and activity in TNBC lines by ancestry. Using Tn5-nicking sites as counts in called peaks, we performed differential ATAC signal analysis using DESeq2. We examined the overabundance of known TF motifs in these differential regions of interest using nonredundant JASPAR motifs and created a heat map of the top 50 most variable TFs (Fig. 2A; Supplementary Table S1). In EA-derived TNBC lines, a strong overabundance of motifs associated with members of the AP-1 TF families was observed, including members of the FOS (FOSL1, FOSL2, FOS), JUN (JUND, JUN, JUNB), and ATF (BATF) and MAF (MAF) subfamilies; AP-1 proteins are bZip domain-containing TFs that homodimerize and heterodimerize with each other and are implicated in cancer cell growth and proliferation across a number of cancer types, including breast (31). One AA-derived cell line, MDA-MB-157, showed a similar pattern, which contrasted with the other four AA cell lines. Differentially accessible chromatin regions (DAR) in the AA-derived TNBC lines showed an overabundance in motifs associated with the Grainyhead family of TFs, such as GRHL1 and TFCP2, which are considered pro-oncogenic in breast cancer and involved in pro-metastatic processes like epithelial-to-mesenchymal transition and angiogenesis (32). AA-associated DARs were also overabundant in motifs of several TFs from the AP-2 family, including AP2A, 2B, and 2C, and multiple isoforms, which have a well-documented role in breast carcinogenesis (33); also enriched were motifs in forkhead box (FOX) TFs, including members of the FOXO4 (implicated in cancer cell invasion and replicative immortality), FOXD2 (implicated in chemoresistance), FOXI1 (associated with poor prognosis in breast cancer), and FOXL1 (modulator of Wnt signaling) subfamilies (34, 35).

Next, we performed a digital footprinting analysis of differentially open chromatin regions using TOBIAS. We uncovered significant differences in TF binding by ancestry (79 TFs with a differential binding score > 0.225 or < −0.225 and FDR < 0.01; Fig. 2B and C; Supplementary Table S2). EA and AA TNBC cell lines exhibited increased binding capacities for 17 and 62 total TFs, respectively, with several common themes shared with the motif enrichment analysis. In EA TNBC, the top 10 differentially bound TFs were all members of subfamilies within the AP-1 TF family. In AA TNBC, the predicted top four differentially bound TFs with an increased binding capacity were all associated with epithelial-to-mesenchymal transition (ZEB1, SNAI1, SNAI2, GRHL2). Others were associated with cancer stemness and chemotherapeutic resistance (TFAP2C, NRF1), cancer cell proliferation (E2F, E2F1, E2F2), and the TP53 pathway (P53, P63, P73). Interestingly, our data also showed increased binding of KAISO in AA TNBC lines (Fig. 2B), whose aberrant expression has been previously linked to a distinct biology and poor outcomes in AA patients with breast and prostate cancer (36–38). We wanted to confirm the correlation between ancestry-specific TF activity identified by ATAC-seq with downstream enrichment of their known transcriptional targets. Using RNA-seq data from our cell lines, we performed GSEA with gene sets from the GTRD to infer key transcriptional regulators based on expression of their known transcriptional targets (Supplementary Table S3). While not all met statistical significance, many TFs that had shown significant enrichment with chromosome accessibility mapping also annotated with GSEA. In EA, two TFs overlapped with the two approaches, RORA (*P*_nom_ = 0.23) and BCL6B (*P*_nom_ = 0.26). In AA, 11 TFs overlapped, including SNAI1 (*P*_nom_ = 0.19), NR1H4 (*P*_nom_ < 0.0001), MSX2 (*P*_nom_ = 0.03), and KLF14 (*P*_nom_ = 0.08). Taken together, these data suggest that TF activities show significant differences by donor ancestry, with African ancestry displaying an ancestry-specific TF activity profile associated with increased aggressiveness in breast cancer.

We next sought to extend these findings from human TNBC cell lines to TNBC patient tumors to investigate if ancestry-specific TF activity showed similar patterning by patient ancestry group. ATAC-seq data were not available within TCGA for validation of TF binding and motif enrichment analyses; however, we leveraged RNA-seq data from triple-negative breast tumor tissues in EA (*n* = 78) and AA (*n* = 34) patients within TCGA to perform an upstream regulator analysis to infer critical transcriptional regulators based on ancestry-related differences in downstream gene expression (Supplementary Table S4). We identified numerous donor ancestry-associated upstream regulators in the tumors that overlapped with the ancestry-related TF activities found in our TNBC cell line analyses (Table 2). In TNBC tumors from EA patients, these TFs totaled 22 and included FOS [activation score (AS) = 1.89, *P* = 0.00033], JUN (AS = 1.90, *P* = 0.00003), JUNB (AS = 0.56, *P* = 0.022), BATF (AS = 2.00, *P* = 0.0035), and FOSL1 (AS = 0.52, *P* = 0.0026). In TNBC tumors from AA patients, validated TFs totaled 25 and notably included TP53 (AS = 0.61, *P* = 0.0093), TP63 (AS = 0.61, *P* = 0.0048), TCF3 (AS = 2.53, *P* < 0.00001) and TCF4 (AS = 1.70, *P* = 0.0001), TFAP2A (AS = 1.3, *P* = 0.004), and various members of the FOX TF family. These patient data were concordant with some of the top TFs identified in our cell line data.

**TABLE 2:** Upstream regulator analysis of TCGA breast cancer data identifies patient group-associated TFs that have a common association with ATAC-seq signals in the TNBC human cell lines

Upstream Regulator Analysis using TCGA RNA-seq Data^a^		
TNBC Tumors from European American Patients (*n* = 78)		
*Enriched upstream regulator*	*Activation z-score*	*P*
ESR1	3.34	0.00000
ETV3	2.24	0.01900
BATF	2.00	0.00350
ETV5	2.00	0.01140
JUN	1.90	0.00003
FOS	1.89	0.00033
MNT	1.89	0.00070
SREBF2	1.83	0.00320
GATA3	1.62	0.01420
SREBF1	1.38	0.00660
ESR2	0.90	0.00155
STAT3	0.87	0.00001
CLOCK	0.71	0.03700
PRDM1	0.68	0.00000
EGR2	0.67	0.00350
JUNB	0.56	0.02200
CUX1	0.56	0.03600
NR4A2	0.54	0.00097
FOSL1	0.52	0.00260
BACH2	0.39	0.01100
EGR1	0.38	0.01260
SRF	0.11	0.01550
**TNBC Tumors from African American Patients (*n* = 34)**		
***Enriched upstream regulator***	***Activation z-score***	***P***
TCF3	2.53	0.00000
MYF6	2.22	0.00686
IRF9	2.19	0.02030
REST	2.19	0.00003
POU2F2	2.12	0.00020
POU4F1	2.00	0.02490
BHLHE40	1.94	0.00214
TBX21	1.83	0.00003
PAX5	1.77	0.00001
TCF4	1.70	0.00001
KLF6	1.56	0.00220
TFAP2A	1.30	0.00400
NR3C2	1.29	0.00487
IRF8	0.89	0.00523
EOMES	0.89	0.00990
TP63	0.68	0.00930
TP53	0.61	0.00484
HOXA10	0.58	0.00290
SMAD3	0.56	0.00000
MSC	0.39	0.00000
SP3	0.37	0.00170
FOXA1	0.22	0.00003
RARA	0.11	0.03070
FOXP3	0.05	0.00112
CTCF	0.05	0.00014

^a^TFs displayed that specifically overlapped with those identified by cell line ATAC analyses.

### TNBC Cells Derived from AA Women Show Downstream Transcriptional Changes Associated More Aggressive Tumor Biology

**FIGURE 3:**
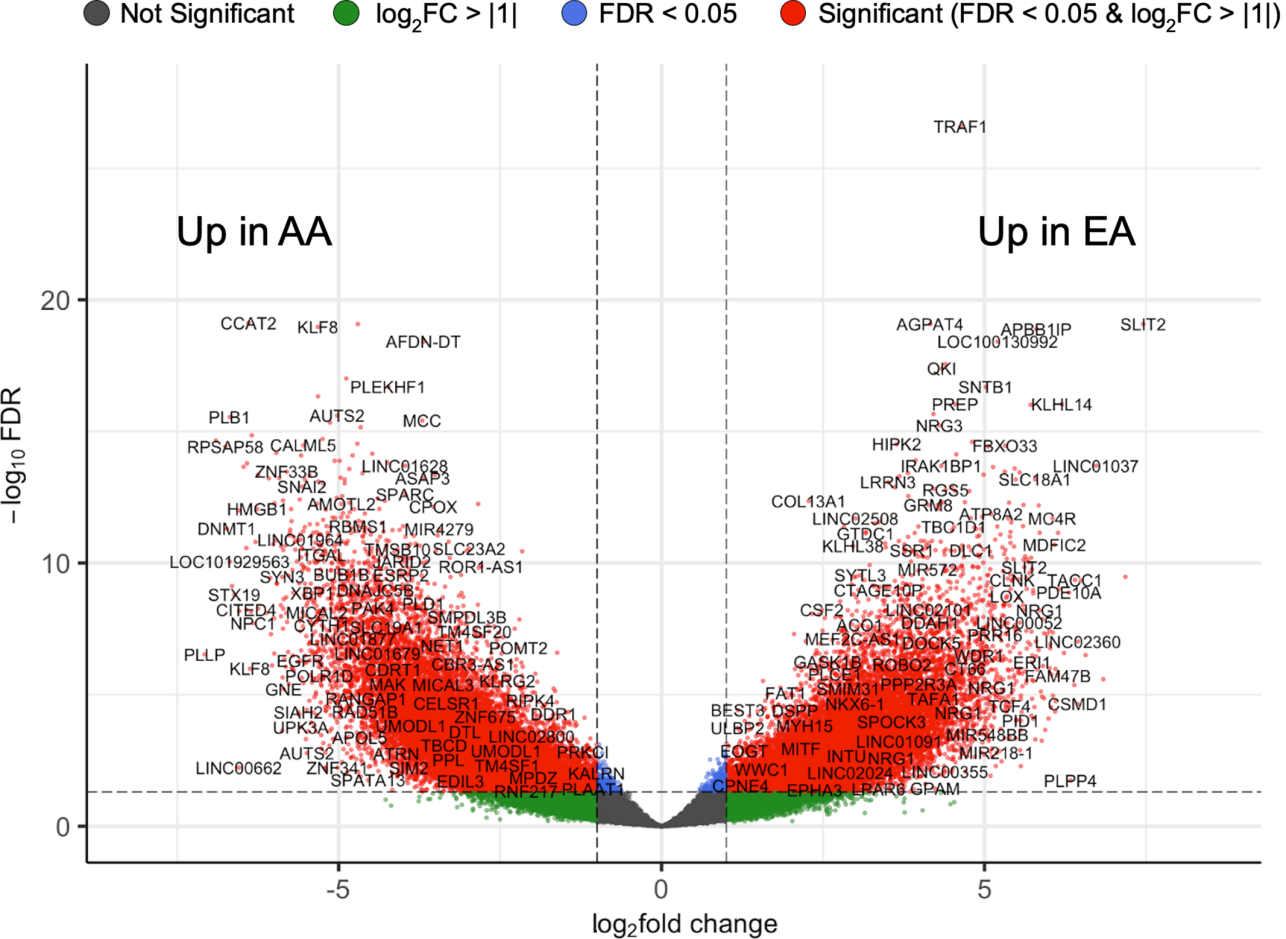
Differential ATAC signal analysis of human TNBC cell lines identified ancestry-associated genes within differentially accessible chromatin regions across all genomic elements. Genes are displayed on a volcano plot, where the *y*-axis shows statistical significance (plotted as −log_10_ FDR) and the *x*-axis shows the differences between each ancestry group (plotted as log_2_ fold change). The horizontal dashed lines depict our specified significance cut-off points, with genes that showed an FDR < 0.05 and a |log_2_FC| > 1 shown in red. Negative log_2_ fold change values show genes differentially open or up in AA donor cell lines, while positive log_2_ fold change values show genes differentially open or up in EA donor cell lines.

Our findings showing that EA and AA women possess differential patterns of TF binding in TNBC cells led us to wonder how these differences may impact downstream gene expression. To this end, we performed a differential ATAC signal analysis using DESeq2 to identify genes within differentially open ATAC-seq peaks, defined as differentially expressed genes or DEGs; |log_2_FC| > 2, FDR < 0.01; Fig. 3; Supplementary Table S5). Across all genomic elements, we identified 13,945 differential peaks in EA TNBC and 14,927 in AA TNBC, mapped to 6,029 and 7,595 unique gene annotations, respectively. Specifically in promoters, we identified 791 genes that were differentially open in EA and 1,596 that were differentially open in AA. Within promoter regions, the top DEG upregulated in EA was *LRRC2* (|log_2_FC| = 5.6, FDR = 6.2 × 10^−13^), a long noncoding RNA suspected to function as a tumor suppressor, followed by *LRIG1*, which interestingly also has an anti-invasive role in basal-like breast cancer cells through encouraging mesenchymal-to-epithelial transition (ref. 39; Table 3). Conversely, the top DEG upregulated in AA was *DNMT1* (|log_2_FC| = 6.73, FDR = 4.9 × 10^−12^), a DNA methyltransferase (Table 3); this epigenetic regulator was also inferred to be enriched in our upstream regulator analysis using GSEA GTRD with our RNA-seq data (ES = 0.30, *P*_nom_ = 0.097; Supplementary Table S3). Interestingly, expression of DNMT1 is associated with poor survival in breast cancer, is overexpressed specifically within TNBC, induces cancer cell invasion and survival through hypermethylation of key tumor suppressors, and is considered an epigenetic target for therapeutic blocking (40). Many of the top-ranking genes identified in differentially accessible chromatin regions by ATAC-seq were also significantly overexpressed at the transcript level in these cell lines (Supplementary Fig. S2; Supplementary Table S6), including top EA-specific DEG, *LRRC2*, and the top AA-specific DEG, *DNMT1*.

**TABLE 3:** Top genes and pathways enriched in TNBC cell lines in association with patient ancestry

Top Genes from Differentially Open Chromatin Regions in EA TNBC				
Number of peaks: 24,699		Number of promoter peaks: 1,939		
Gene	Gene name	Gene region	log_2_ Fold change	FDR
LRRC2-AS1	LRRC2 antisense RNA 1	Promoter	5.602271229	6.20E-13
LRIG1	Leucine-rich repeats and immunoglobulin-like domains 1	Promoter	5.595517478	4.00E-12
WHAMMP1	WAS protein homolog associated with actin, golgi membranes, and microtubules pseudogene 1	Promoter	5.385911582	4.00E-04
DAAM2	Disheveled-associated activator of morphogenesis 2	Promoter	5.371681742	1.20E-06
FIG4	FIG4 phosphoinositide 5-phosphatase	Promoter	5.161573716	4.80E-09
ZNF175	Zinc finger protein 175	Promoter	5.114722072	5.60E-09
ANKS1B	Ankyrin repeat and sterile alpha motif domain containing 1B	Promoter	5.032588901	1.50E-07
SSH1	Slingshot protein phosphatase 1	Promoter	4.917551442	9.00E-12
TNFSF10	TNF superfamily member 10	Promoter	4.900819915	3.00E-04
SSB	Small RNA-binding exonuclease protection factor La	Promoter	4.878610195	1.00E-04
**Top Enriched Pathways from Differentially Open Chromatin Regions in EA TNBC (FDR < 0.2)**				
**Top enriched cancer hallmark pathways**	**Adjusted *P* value**	**Pathway enrichment score**		
Epithelial–mesenchymal transition	2.30E-04	39.58		
Hedgehog signaling	0.16	17.08		
UV response Dn	0.044	16.81		
Complement	0.1	10.9		
**Top Genes from Differentially Open Chromatin Regions in AA TNBC**				
**Number of peaks: 26,481**		**Number of promoter peaks: 3,676**		
**Gene**	**Gene name**	**Gene region**	**log_2_ Fold change**	**FDR**
DNMT1	DNA methyltransferase 1	Promoter	−6.7310574	4.93E-12
STX19	Syntaxin 19	Promoter	−6.616313	1.60E-09
RBBP8	RB binding protein 8, endonuclease	Promoter	−6.5506956	1.00E-12
BDH1	3-hydroxybutyrate dehydrogenase 1	Promoter	−6.3479744	1.40E-15
LINC01143	Long intergenic non-protein coding RNA 1143	Promoter	−6.2482495	5.32E-09
LINC00536	Long intergenic non-protein coding RNA 536	Promoter	−5.9931338	5.33E-13
CLDN8	Claudin 8	Promoter	−5.9201921	5.88E-14
PDE9A	Phosphodiesterase 9A	Promoter	−5.8916671	1.04E-08
PTH2R	Parathyroid hormone 2 receptor	Promoter	−5.7741998	3.01E-10
TBCD	Tubulin folding cofactor D	Promoter	−5.677859901	1.55E-11
**Top Enriched Pathways from Differentially Open Chromatin Regions in AA TNBC (FDR < 0.2)**				
**Top enriched cancer hallmark pathways**	**Adjusted *P* value**	**Pathway enrichment score**		
Estrogen response early	4.10E-04	28.99		
Apical junction	2.90E-03	20.06		
Estrogen response late	4.40E-04	17.56		
Hypoxia	1.10E-01	8.11		
Myogenesis	1.60E-01	6.77		
**Top enriched KEGG pathways**	**Adjusted *P* value**	**Pathway enrichment score**		
Vascular smooth muscle contraction	5.16E-04	41.32		
alpha-Linolenic acid metabolism	1.20E-02	61.42		
Fatty acid biosynthesis	2.20E-02	59.88		
Linoleic acid metabolism	2.20E-02	42.36		
GnRH signaling pathway	6.10E-02	17.92		
Fc gamma R-mediated phagocytosis	8.20E-02	15.83		
Platelet activation	8.20E-02	13.98		
Estrogen signaling pathway	1.10E-01	12.34		
Inflammatory mediator regulation of TRP channels	1.50E-01	12.19		

To identify key signaling pathways associated with donor ancestry, we next performed an overrepresentation analysis using the top 500 significant DEGs (|log_2_FC| > 2, FDR < 0.01) upregulated in promoter regions. In EA TNBC, enriched Hallmark-annotated cancer pathways (FDR < 0.2) included epithelial-to-mesenchymal transition (*P*_adjusted_ = 2.3 × 10^−4^), downregulation of UV response (*P*_adjusted_ = 0.04), complement (*P*_adjusted_ = 0.1), and hedgehog signaling (*P*_adjusted_ = 0.16; Table 3). Interestingly, the key DEGs contributing to the enrichment of the epithelial-to-mesenchymal transition pathway (e.g., *fibrillin-1*, *SLIT2*, *WIPF1*, etc.) are negative regulators of this process (41–43), suggesting that this may in fact functionally translate to suppression of mesenchymal differentiation in EA TNBC. No significant enrichment of KEGG-annotated pathways was found. In AA TNBC, upregulated Hallmark-annotated cancer pathways (FDR < 0.2) included estrogen response (early, *P*_adjusted_ = 4.1 × 10^−4^; late, *P* = 4.4 × 10^−4^), apical junction (*P*_adjusted_ = 2.9 × 10^−3^), and hypoxia (*P*_adjusted_ = 0.1), among others (Table 3). Nine KEGG pathways were significantly upregulated in AA TNBC, including angiogenic, metabolic, and inflammatory pathways, for example, inflammatory mediator regulation of transient receptor potential (TRP) channels. Contributing to the enrichment of the TRP pathway was the upregulation of several members of the TRP calcium ion channel superfamily, including *TRPM2*, *TRPM4*, *TRPV6*, and *TRPS1*, in AA TNBC. TRP channels and their regulation of Ca^2+^ signaling have been implicated in cancer proliferation, metastasis, and drug resistance, and are just emerging as a novel candidate for therapeutic intervention in breast cancer (44). Together, these data suggest an enrichment of genes and pathways specific to donor ancestry, with AA-derived TNBC lines showing changes consistent with a more generally aggressive tumor biology.

### Hypoxia Exacerbates Underlying Differences in TF Activity Between EA- and AA-derived TNBC Cell Lines

**FIGURE 4:**
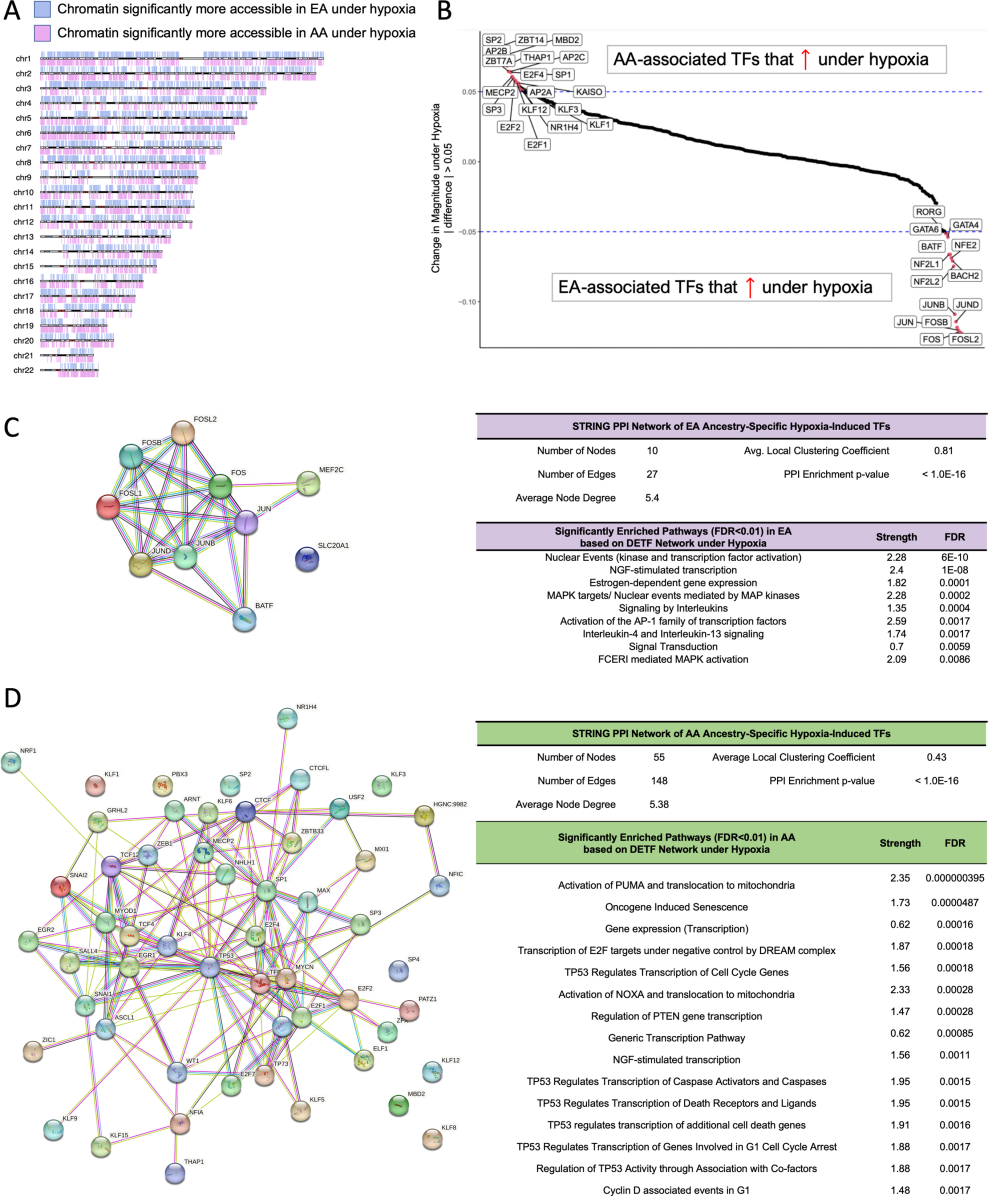
Hypoxia exacerbates donor ancestry-related differences in TF activity in cultured TNBC cell lines. **A,** Chromosome map showing the distribution of DARs between EA and AA TNBC cells when subjected to hypoxic conditions (FC > 2, FDR < 0.01). **B,** Differential digital footprinting in EA- and AA-derived TNBC lines was performed using TOBIAS to identify open chromatin regions and their TF binding sites. Shown are the top AA- or EA-associated TFs whose predicted binding activity increased by a defined magnitude of >0.05 under hypoxia. PPI network of TFs with differential binding capacities (DBS > 22.5, FDR < 0.01) that are up in EA (**C**) and AA (**D**) under hypoxia using the STRING database. Top 15 significantly enriched pathways (FDR < 0.01, Reactome-based) based on a TF network analysis.

Hypoxia, or regions that lack sufficient oxygen, occurs in most solid tumors, including breast cancer. Of all breast cancer subtypes, hypoxia occurs most frequently in TNBC, and has been associated with therapy resistance and poor prognosis (45, 46). Furthermore, a recent study by Bassiouni and colleagues found hypoxic tumor content differed by patient race in TNBC (47). Given the distinct molecular characteristics, we have uncovered thus far in AA TNBC, including an enrichment for the hypoxia pathway at baseline, we wondered how this might impact their biological response to hypoxia. To this end, we cultured EA- and AA-derived TNBC cell lines under normoxic (21% O_2_) and hypoxic (1% O_2_) conditions and performed ATAC-seq. Genome-wide analysis of differentially accessible chromatin sites (FDR < 0.01 and |log_2_FC| > 2) in EA compared with AA TNBC under hypoxia revealed distinctive patterning of open chromatin across a number of chromosomes (Fig. 4A). To look at TF binding, we performed a digital footprinting analysis of differentially open chromatin regions using TOBIAS. While the most differentially expressed transcription factors (DETF) showed changes in the same general direction when comparing EA with AA under both normoxia and hypoxia, the magnitude of these changes showed variations; most hovered near zero, but a core set of TFs within each donor group deviated more dramatically (Fig. 4B; Supplementary Table S7). In EA TNBC, this core set of EA-specific hypoxia response TFs once again included members of the AP-1 TF family, which have been found to be induced in hypoxic environments (48), as well as NFE2 and NF2L1/2 which have been linked to Wnt signaling in breast cancer (49). In AA TNBC, this core set of AA-specific hypoxia response TFs includes six members of the large multigene family of Sp/Kruppel-like factor (KLF) TFs, all of which have been linked to altered cancer cell metabolism; further, three members of the Sp subfamily (SP1, SP2, SP3) have been shown to regulate hypoxia through direct interaction with HIF1a (50). Also, among this core set of AA-specific hypoxia response TFs was KAISO, which has been shown to regulate HIF1a specifically during hypoxia (51) and whose aberrant activity is heavily associated with tumors from AA patients.

To better understand how differentially bound TFs involved in the ancestry-driven hypoxia response might act in concert to dictate downstream gene expression, we constructed a protein-protein interaction (PPI) network using the STRING database (27), allowing for better discernment of functional relationships between TFs (Fig. 4C and D). Within the core set of EA-specific hypoxia-induced TFs, a significantly enriched PPI network was observed (*P* < 1.0 × 10^−16^), with a high degree of TF coexpression and shared functions (average local clustering coefficient = 0.81) among the small network (nodes, *n* = 10, edges, *n* = 27); as expected, DETFs from AP-1 family, namely the JUN and FOS TF subfamilies, showed highly robust interactions, likely reflecting the homodimeric and heterodimeric functional relationships between these proteins. We performed a pathway enrichment analysis using REACTOME based on the TF network analysis to identify shared biologic function of significant TF clusters (Fig. 4C); the top FDR-significant pathways included the pathways involved in proliferation (MAPK targets; FCERI-mediated MAPK activation), anti-inflammatory cytokine signaling (IL4 and IL13 signaling; signaling by ILs), and activation of the AP-1 family of TFs. Within the core set of AA-specific hypoxia-induced TFs, a significantly enriched PPI network was also observed (*P* < 1.0 × 10^−16^), which was more expansive and extensive than that of the EA PPI network (nodes, *n* = 55, edges, *n* = 148). TP53 is one of the most prominent nodes featured in this network, with high degree of connectivity and central to several other key nodes, such as SP-1, EGR-1, and KLF-4. In pathway enrichment analyses using REACTOME, 33 pathways were identified with an FDR < 0.01, 15 of which are shown in Fig. 4D. Top pathway themes in the AA TF network were many pathways related to TP53, cell cycle (E2F; cyclin D and G1; PTEN; transcription of E2F targets; TP53 regulation transcription of cell-cycle genes; etc.), and cell stress/apoptosis (activation of PUMA; activation of NOXA; oncogene-induced senescence).

We next performed a differential ATAC signal analysis using DESeq2 to better understand downstream genes that may be impacted by the increased activity of each of the ancestry-specific hypoxia response TFs (Table 4; Supplementary Table S8). Across all genomic elements, we identified 5,643 differential peaks in EA TNBC and 7,037 in AA TNBC, mapped to 3,595 and 4,618 unique gene annotations, respectively. Specifically in promoters, we identified 491 genes that were differentially accessible in EA and 1,001 that were differentially accessible in AA, with an increased access to them for TFs. Within promoter regions, the top DEGs upregulated in EA was *MAGI1*, a junctional scaffold protein that acts as a tumor suppressor in the context breast cancer through inhibition of the p38 stress pathway (52), and *SMG6*, which has been implicated in DNA repair and telomere maintenance (53). The top DEGs upregulated in AA was endoplasmic reticulum-associated protein *ZDHHC1*, implicated in innate immune response and a positive regulator of the STING pathway (54), and *ADAP1*, which has been shown to promote cancer progression by inducing cell migration and invasion (55). All together, these data suggest that TNBC cells may have distinct, donor ancestry-specific stress responses to hypoxia.

**TABLE 4:** Top DEGs upregulated under hypoxia in EA and AA TNBC cell lines

Top 10 DEGs from DARs in Promoter Regions Upregulated in EA under Hypoxia			
Symbol	Gene	log_2_ Fold change	FDR
MAGI1	Membrane-associated guanylate kinase, WW and PDZ domain containing 1	4.68	3.02E-12
SMG6	SMG6 nonsense-mediated mRNA decay factor	4.58	6.57E-07
DAAM2	Disheveled-associated activator of morphogenesis 2	4.51	9.73E-05
DPP9	Dipeptidyl peptidase 9	4.49	7.52E-07
CDCA2	Cell division cycle associated 2	4.41	2.17E-22
SSH1	Slingshot protein phosphatase 1	4.12	4.10E-07
C3orf18	Chromosome 3 open reading frame 18	4.08	3.05E-11
ITPR2	Inositol 1,4,5-trisphosphate receptor type 2	4	5.43E-07
FRMD6	FERM domain containing 6	3.99	6.47E-06
KRTAP2–3	Keratin-associated protein 2–3	3.98	5.28E-07
**Top 10 DEGs from DARs in Promoter Regions Upregulated in AA under Hypoxia**			
**Symbol**	**Gene**	**log_2_ Fold change**	**FDR**
ZDHHC1	Zinc finger DHHC-type containing 1	−6.08	1.11E-14
ADAP1	ArfGAP with dual PH domains 1	−6.04	1.70E-16
CRYBG2	Crystallin beta-gamma domain containing 2	−5.73	1.97E-12
CRISPLD2	Cysteine rich secretory protein LCCL domain containing 2	−5.73	1.67E-12
MB	Myoglobin	−5.72	6.47E-13
CBLC	Cbl proto-oncogene C	−5.7	8.52E-09
SPECC1L-ADORA2A	SPECC1L-ADORA2A readthrough (NMD candidate)	−5.67	2.36E-13
C1orf116	Chromosome 1 open reading frame 116	−5.61	2.41E-15
KIFC3	Kinesin family member C3	−5.59	1.12E-10
CNKSR1	Connector enhancer of kinase suppressor of Ras 1	−5.58	1.13E-13

## Discussion

Here, we adopted a reductionist approach to better understand the contribution of patient ancestry to TNBC at the chromosomal level. In this study, we uncovered an ancestry-specific chromatin landscape across nine commonly used human TNBC cell lines. Through chromatin profiling by ATAC-seq, we found that TNBC cell lines displayed separation of chromatin profiles by donor ancestry. ATAC-seq is uniquely positioned to explore TF activity and binding; therefore, we were especially interested in characterizing ancestry-specific TFs, as these proteins are master regulators of chromatin and downstream gene expression and their dysregulation is frequently implicated in cancer initiation and progression.

EA-derived TNBC lines showed an overabundance in motifs associated with members of AP-1 TF families that are linked to cancer cell growth and proliferation. AA-derived TNBC lines displayed strong overabundance in motifs associated with members of the Grainyhead, Forkhead Box, and AP-2 TF, which have been extensively linked to a pro-oncogenic and pro-metastatic tumor biology, notably including cancer cell invasion, epithelial-to-mesenchymal transition, angiogenesis, and poor prognosis in breast cancer. Digital footprinting analysis identified differentially bound TFs, which suggests activity at the time of the assay and may be of particular functional relevance, further bolstered these findings. Indeed, EA and AA TNBC cell lines exhibited increased binding of 17 and 62 total TFs, respectively. Particularly interesting were the differentially bound TFs specific to African ancestry, of which a majority had robust links to epithelial-to-mesenchymal transition, as well as many associated with cancer stemness, chemotherapeutic resistance, cancer cell proliferation, and the p53 pathway. Notably, we found increased binding of KAISO in AA TNBC lines. Several studies have shown KAISO to be highly and preferentially expressed in TNBC tissues of women of African descent (56, 57). In both breast and prostate cancer, KAISO promotes cell migration and invasion through silencing of methylated genes that promote epithelial-to-mesenchymal transition (38). Indeed, its aberrant expression has been previously linked to a distinct biology and poor outcomes in individuals of African descent (57). Our study further corroborates these findings at the chromatin level, showing ancestry-specific binding of this critical and multifunctional transcriptional regulator. We performed validation of these findings in human patients, identifying a number of upstream transcriptional regulators enriched in TNBC tumors from TCGA that overlapped with those found in our cell lines, which included 22 shared between patient tumors and cell lines of predominantly European ancestry, and 25 shared between patient tumors and cell lines of predominantly African ancestry; these TFs may be of particular interest for future follow-up studies. Overall, we observed an African ancestry-related TF profile in TNBC that is largely associated with a more aggressive and pro-metastatic tumor biology.

Upon examining how these ancestry-specific TF profiles could impact gene expression, particularly of those that fell within promoter regions, we identified a number of interesting candidate genes upregulated in TNBC cell lines of African ancestry, including *DNMT1*. A DNA methyltransferase, *DNMT1* expression has been shown to be overexpressed specifically in TNBC associated with poor survival (40). It functions include a repression of estrogen receptor expression and signaling, promotion of epithelial-to-mesenchymal transition, activation of cellular autophagy, and cancer stem cell growth, achieving these functions through the hypermethylation of the estrogen receptor promoter region, tumor suppressor genes, and other cancer progression inhibiting factors (40). DNMT1 inhibitors have shown antitumorigenic activity and enhance sensitivity to immunotherapies. Our findings suggest that targeting this candidate gene may have preferential benefit for patients of African descent and more extensive follow-up studies are warranted to better understand its ancestry-specific role. Pathway enrichment analyses identified numerous significantly enriched pathways upregulated in AA TNBC lines, including those involved in cancer metabolism, inflammation, and cell adhesion. One particularly interesting finding was the enrichment of inflammatory mediator regulation of TRP channels specific to African ancestry, driven in part by the upregulation of several members of the TRP calcium ion channel superfamily, including *TRPM2*, *TRPM4*, *TRPV6*, and *TRPS1*. TRP channels and their regulation of Ca^2+^ signaling have been shown to induce cancer proliferation, metastasis, and drug resistance. Accordingly, they are emerging as novel candidates for therapeutic targeting; yet their connection to African ancestry was previously unknown prior to these findings, which may now warrant further investigation to evaluate their potential for enhanced efficacy in AA women with breast cancer. While it was perhaps counterintuitive to find enriched pathways in estrogen response, closer examination of the genes responsible for driving that overrepresentation have functions outside of estrogen response and known roles in TNBC, including *SLC9A1*, which is implicated in epithelial-to-mesenchymal transition in TNBC (58), *FAM102A*, which is differentially expressed in BRCA-mutated cancers (59) that tend to be triple-negative, and *CDH1*, which is linked to TNBC proliferation and invasion (60). Furthermore, AA-associated DEGs driving the putative estrogen response pathway included several genes associated with cellular junctions, such as *ZO-3/TJP3*, *CELSR3*, *claudin-7*, which is overexpressed in TNBC and associated with worse outcomes (61).

Of interest, one upregulated pathway related to African ancestry was hypoxia, which commonly develops in TNBC and may vary in its extent by ancestral background, as suggested recently (47). This prompted us to further explore how TNBC cells of differing ancestral backgrounds responded to hypoxic conditions. This led us to identify a core set of differentially bound ancestry-specific TFs exacerbated under hypoxia. A tight network with high TF coexpression emerged in EA-derived TNBC, with enrichment of MAPK signaling and several anti-inflammatory cytokines. In TNBC of African ancestry, a large, extensive TF network with numerous nodes and connections was constructed. Among the many significantly enriched pathways related to these hypoxia-induced TFs, common themes of oxidative stress, DNA damage, apoptosis, and cell cycle emerged, with p53 appearing as a central node. Upon examination of top DEGs from DARs in promoter regions, top genes specific to EA-derived TNBC cells were associated with tumor suppressive and antiproliferative roles, while top genes specific to AA-derived TNBC cells were implicated in STING-driven immune response and cell migration/invasion. When taken together, these data not only enhances our understanding of ancestry-specific hypoxia response, but at a broader level may also suggest that the underlying molecular differences in TNBC by genetic ancestry could contribute to distinct response to other experimental manipulations and perturbations. This could have implications for mechanistic work, drug discovery, response to therapy, and other work based on *in vitro* models, thereby further underscoring that donor ancestry should be considered in experimental design.

Understanding the contribution of genetic ancestry to breast cancer disparities in disease aggressiveness and outcomes is a current research priority in the field. Work championed by several investigators in this field has moved the needle considerably in evaluating African ancestry as a true biologic determinant of TNBC separate from race (6, 7, 62, 63). Indeed, several notable studies have identified ancestry-related gene signatures in human TNBC tumors (7, 63, 64), which were largely dominated by differential immune cell infiltrates and activation. Here, we explore this important scientific question through the lens of epigenetic, rather than transcriptional regulation. This gives us the opportunity to expand our understanding of genetic ancestry by looking at the upstream master regulators of gene expression—TFs—which were central to our investigation. Our findings have shown that African ancestry in TNBC is associated with the distinct activity of critical TFs and downstream gene expression changes that may contribute in part to observed disparities in tumor aggressiveness in this population group. Furthermore, our study focuses on human cell lines, rather than patient tumors, to explore genetic ancestry at the molecular level independent of common confounders that are inextricably linked to human cohorts, such as socioeconomic status, access to quality health care, comorbidities, lifestyle factors, and other socioenvironmental factors that play central roles to cancer disparities, thereby allowing this *in vitro*-based study to further bolster existing evidence in the field that genetic ancestry may contribute to worsened TNBC biology.

Finally, the findings from this study may have broader implications beyond TNBC. Human cancer cell lines are foundational to basic science discovery, drug development, and preclinical research. The cell lines selected in this study represent common breast cancer lines used routinely in cancer research at the bench. We found that the cellular behavior and response to experimental conditions can vary as a result of ancestral origin. This suggests that the ancestral origin of patient-derived cell lines matters and the development and use of diversely sourced cell lines should be considered in experimental design in *in vitro* studies. Not only can this approach improve study rigor by reducing erroneous biologic variation between studies, but it also represents a step toward improving inclusivity and increasing health equity even at this most basic level in biomedical research.

## Supplementary Material

Supplementary Figure 1 Supplemental Figure 1. Quantified genetic ancestry in TCGA participants by self-reported race. Using published ancestry annotations from Carrot-Zhang et al1, quantified genetic ancestry was compared to self-reported race (SRR). For 111/112 TCGA samples used in our study, genetic ancestry consensus was concordant with SRR. Genetic ancestry consensus classification of African or admixed African ancestry captured 100% of TCGA participants who self-identified as “Black or African American”. Genetic ancestry consensus classification of EUR ancestry captured 98.7% of TCGA participants who self-identified as “White”; in one case, a participant that self-identified as White demonstrated a higher proportion of admixed American genetic ancestry. Among TCGA participants who self-identified as Black or African American, the average percentage of African ancestry was 83.2%, which is comparable to the quantified genetic ancestry estimates for the human cell lines used in this study that were classified as African American-derived (average African ancestry of 79.5%). Among TCGA participants who self-identified as White, the average percentage of European ancestry was 96.7%, which is comparable to the quantified genetic ancestry estimates for the human cell lines used in this study that were classified as European American-derived (average European ancestry of 93.0%).

Supplementary Figure 2 Supplemental Figure 2. Expression of genes by donor ancestry in TNBC cell lines. Top-ranked genes identified to be located in open chromatin regions with an ancestry relationship were individually confirmed to be differentially expressed at the transcript level in matched RNA-seq data from the same human TNBC cell lines. Significance testing with two-sided t-test.

Supplementary Table 1 Supplementary table 1 displaying data output from motif enrichment analysis performed on ATAC-seq data from TNBC cell lines.

Supplementary Table 2 Supplementary table 2 displaying data output from digital footprinting analysis performed on ATAC-seq data from TNBC cell lines.

Supplementary Table 3 Supplementary table 3 displaying data output from gene set enrichment analysis for transcriptional regulators performed on RNA-seq data from TNBC cell lines.

Supplementary Table 4 Supplementary table 4 displaying data output from upstream regulator analysis performed on RNA-seq data from TNBC patients in TCGA.

Supplementary Table 5 Supplementary table 5 displaying data output from differential ATAC signal performed on ATAC-seq data from TNBC cell lines.

Supplementary Table 6 Supplementary table 6 displaying data output from differential gene expression analysis performed on RNA-seq data from TNBC cell lines.

Supplementary Table 7 Supplementary table 7 displaying data output from digital footprinting analysis performed on ATAC-seq data from TNBC cell lines cultured under hypoxia.

Supplementary Table 8 Supplementary table 8 displaying data output from differential ATAC signal analysis performed on ATAC-seq data from TNBC cell lines cultured under hypoxia.

## References

[1] Mirabelli P , Coppola L, Salvatore M. Cancer cell lines are useful model systems for medical research. Cancers 2019;11:1098. 31374935 10.3390/cancers11081098 PMC6721418

[2] Hooker SE , Woods-Burnham L, Bathina M, Lloyd S, Gorjala P, Mitra R, . Genetic ancestry analysis reveals misclassification of commonly used cancer cell lines. Cancer Epidemiol Biomarkers Prev 2019;28:1003–9. 30787054 10.1158/1055-9965.EPI-18-1132 PMC6548687

[3] Woods-Burnham L , Basu A, Cajigas-Du Ross CK, Love A, Yates C, De Leon M, . The 22Rv1 prostate cancer cell line carries mixed genetic ancestry: implications for prostate cancer health disparities research using pre-clinical models. Prostate 2017;77:1601–8. 29030865 10.1002/pros.23437 PMC5687283

[4] AACR. Cancer Disparities Progress Report 2022; 2022. Available from: http://www.CancerDisparitiesProgressReport.org/. 10.1158/1055-9965.EPI-22-0542 35675281

[5] Jiagge E , Oppong JK, Bensenhaver J, Aitpillah F, Gyan K, Kyei I, . Breast cancer and African ancestry: lessons learned at the 10-year anniversary of the ghana-michigan research partnership and international breast registry. J Glob Oncol 2016;2:302–10. 28717716 10.1200/JGO.2015.002881 PMC5493263

[6] Lord BD , Martini RN, Davis MB. Understanding how genetic ancestry may influence cancer development. Trends Cancer 2022;8:276–9. 35027335 10.1016/j.trecan.2021.12.006

[7] Martini R , Delpe P, Chu TR, Arora K, Lord B, Verma A, . African ancestry–associated gene expression profiles in triple-negative breast cancer underlie altered tumor biology and clinical outcome in women of African descent. Cancer Discov 2022;12:2530–51. 36121736 10.1158/2159-8290.CD-22-0138 PMC9627137

[8] Corces MR , Trevino AE, Hamilton EG, Greenside PG, Sinnott-Armstrong NA, Vesuna S, . An improved ATAC-seq protocol reduces background and enables interrogation of frozen tissues. Nat Methods 2017;14:959–62. 28846090 10.1038/nmeth.4396 PMC5623106

[9] Caravaca JM , Mehta M, Gowda S, Tran B. ATAC sequencing protocol for cryopreserved mammalian cells. Bio Protoc 2022;12:e4294. 10.21769/BioProtoc.4294 PMC8799669 35127984

[10] Martin M . Cutadapt removes adapter sequences from high-throughput sequencing reads. EMBnet J 2011;17:10–12.

[11] Langmead B , Salzberg SL. Fast gapped-read alignment with Bowtie 2. Nat Methods 2012;9:357–9. 22388286 10.1038/nmeth.1923 PMC3322381

[12] ENCODE ATAC-seq pipeline. Available from: https://www.encodeproject.org/atac-seq/.

[13] Genrich: detecting sites of genomic enrichment. Available from: https://github.com/jsh58/Genrich.

[14] FastQC: a quality control tool for high throughput sequence data. Available from: https://www.bioinformatics.babraham.ac.uk/projects/fastqc/.

[15] Daley T , Smith AD. Predicting the molecular complexity of sequencing libraries. Nat Methods 2013;10:325–7. 23435259 10.1038/nmeth.2375 PMC3612374

[16] Ou J , Liu H, Yu J, Kelliher MA, Castilla LH, Lawson ND, . ATACseqQC: a Bioconductor package for post-alignment quality assessment of ATAC-seq data. BMC Genomics 2018;19:169. 29490630 10.1186/s12864-018-4559-3 PMC5831847

[17] Corces MR , Granja JM, Shams S, Louie BH, Seoane JA, Zhou W, . The chromatin accessibility landscape of primary human cancers. Science 2018;362:eaav1898. 30361341 10.1126/science.aav1898 PMC6408149

[18] Liao Y , Smyth GK, Shi W. featureCounts: an efficient general purpose program for assigning sequence reads to genomic features. Bioinformatics 2014;30:923–30. 24227677 10.1093/bioinformatics/btt656

[19] Yan F , Powell DR, Curtis DJ, Wong NC. From reads to insight: a hitchhiker's guide to ATAC-seq data analysis. Genome Biol 2020;21:22. 32014034 10.1186/s13059-020-1929-3 PMC6996192

[20] Yu G , Wang LG, He QY. ChIPseeker: an R/Bioconductor package for ChIP peak annotation, comparison and visualization. Bioinformatics 2015;31:2382–3. 25765347 10.1093/bioinformatics/btv145

[21] Love MI , Huber W, Anders S. Moderated estimation of fold change and dispersion for RNA-seq data with DESeq2. Genome Biol 2014;15:550. 25516281 10.1186/s13059-014-0550-8 PMC4302049

[22] Schep AN , Wu B, Buenrostro JD, Greenleaf WJ. chromVAR: inferring transcription factor-associated accessibility from single-cell epigenomic data. Nat Methods 2017;14:975–8. 28825706 10.1038/nmeth.4401 PMC5623146

[23] Castro-Mondragon JA , Riudavets-Puig R, Rauluseviciute I, Berhanu Lemma R, Turchi L, Blanc-Mathieu R, . JASPAR 2022: the 9th release of the open-access database of transcription factor binding profiles. Nucleic Acids Res 2022;50:D165–73. 34850907 10.1093/nar/gkab1113 PMC8728201

[24] Kulakovskiy IV , Vorontsov IE, Yevshin IS, Sharipov RN, Fedorova AD, Rumynskiy EI, . HOCOMOCO: towards a complete collection of transcription factor binding models for human and mouse via large-scale ChIP-Seq analysis. Nucleic Acids Res 2018;46:D252–9. 29140464 10.1093/nar/gkx1106 PMC5753240

[25] Bentsen M , Goymann P, Schultheis H, Klee K, Petrova A, Wiegandt R, . ATAC-seq footprinting unravels kinetics of transcription factor binding during zygotic genome activation. Nat Commun 2020;11:4267. 32848148 10.1038/s41467-020-18035-1 PMC7449963

[26] Chen EY , Tan CM, Kou Y, Duan Q, Wang Z, Meirelles GV, . Enrichr: interactive and collaborative HTML5 gene list enrichment analysis tool. BMC Bioinformatics 2013;14:128. 23586463 10.1186/1471-2105-14-128 PMC3637064

[27] Szklarczyk D , Gable AL, Lyon D, Junge A, Wyder S, Huerta-Cepas J, . STRING v11: protein–protein association networks with increased coverage, supporting functional discovery in genome-wide experimental datasets. Nucleic Acids Res 2019;47:D607–13. 30476243 10.1093/nar/gky1131 PMC6323986

[28] Carrot-Zhang J , Chambwe N, Damrauer JS, Knijnenburg TA, Robertson AG, Yau C, . Comprehensive analysis of genetic ancestry and its molecular correlates in cancer. Cancer Cell 2020;37:639–54. 32396860 10.1016/j.ccell.2020.04.012 PMC7328015

[29] Grandi FC , Modi H, Kampman L, Corces MR. Chromatin accessibility profiling by ATAC-seq. Nat Protoc 2022;17:1518–52. 35478247 10.1038/s41596-022-00692-9 PMC9189070

[30] Bushweller JH . Targeting transcription factors in cancer — from undruggable to reality. Nat Rev Cancer 2019;19:611–24. 31511663 10.1038/s41568-019-0196-7 PMC8820243

[31] Wu Z , Nicoll M, Ingham RJ. AP-1 family transcription factors: a diverse family of proteins that regulate varied cellular activities in classical hodgkin lymphoma and ALK+ ALCL. Exp Hematol Oncol 2021;10:4. 33413671 10.1186/s40164-020-00197-9 PMC7792353

[32] Kotarba G , Krzywinska E, Grabowska AI, Taracha A, Wilanowski T. TFCP2/TFCP2L1/UBP1 transcription factors in cancer. Cancer Lett 2018;420:72–9. 29410248 10.1016/j.canlet.2018.01.078

[33] Thewes V , Orso F, Jäger R, Eckert D, Schäfer S, Kirfel G, . Interference with activator protein-2 transcription factors leads to induction of apoptosis and an increase in chemo- and radiation-sensitivity in breast cancer cells. BMC Cancer 2010;10:192. 20459791 10.1186/1471-2407-10-192 PMC2890516

[34] Bach DH , Long NP, Luu TTT, Anh NH, Kwon SW, Lee SK. The dominant role of forkhead box proteins in cancer. Int J Mol Sci 2018;19:3279. 30360388 10.3390/ijms19103279 PMC6213973

[35] Onodera Y , Takagi K, Neoi Y, Sato A, Yamaguchi M, Miki Y, . Forkhead box I1 in breast carcinoma as a potent prognostic factor. Acta Histochem Cytochem 2021;54:123–30. 34511651 10.1267/ahc.21-00034 PMC8424250

[36] Jones J , Wang H, Karanam B, Theodore S, Dean-Colomb W, Welch DR, . Nuclear localization of Kaiso promotes the poorly differentiated phenotype and EMT in infiltrating ductal carcinomas. Clin Exp Metastasis 2014;31:497–510. 24570268 10.1007/s10585-014-9644-7 PMC4065802

[37] Jones J , Wang H, Zhou J, Hardy S, Turner T, Austin D, . Nuclear Kaiso indicates aggressive prostate cancers and promotes migration and invasiveness of prostate cancer cells. Am J Pathol 2012;181:1836–46. 22974583 10.1016/j.ajpath.2012.08.008 PMC3483816

[38] Wang H , Liu W, Black S, Turner O, Daniel JM, Dean-Colomb W, . Kaiso, a transcriptional repressor, promotes cell migration and invasion of prostate cancer cells through regulation of miR-31 expression. Oncotarget 2015;7:5677–89. 10.18632/oncotarget.6801 PMC4868713 26734997

[39] Yokdang N , Hatakeyama J, Wald JH, Simion C, Tellez JD, Chang DZ, . LRIG1 opposes epithelial-to-mesenchymal transition and inhibits invasion of basal-like breast cancer cells. Oncogene 2016;35:2932–47. 26387542 10.1038/onc.2015.345 PMC4805527

[40] Wong KK . DNMT1: a key drug target in triple-negative breast cancer. Semin Cancer Biol 2021;72:198–213. 32461152 10.1016/j.semcancer.2020.05.010

[41] Xin W , Zhao C, Jiang L, Pei D, Zhao L, Zhang C. Identification of a novel epithelial–mesenchymal transition gene signature predicting survival in patients with HNSCC. Pathol Oncol Res 2021;27:585192. 34257533 10.3389/pore.2021.585192 PMC8262154

[42] Ahirwar DK , Charan M, Mishra S, Verma AK, Shilo K, Ramaswamy B, . Slit2 inhibits breast cancer metastasis by activating M1-like phagocytic and antifibrotic macrophages. Cancer Res 2021;81:5255–67. 34400395 10.1158/0008-5472.CAN-20-3909 PMC8631742

[43] Lien HC , Lee YH, Juang YL, Lu YT. Fibrillin-1, a novel TGF-beta-induced factor, is preferentially expressed in metaplastic carcinoma with spindle sarcomatous metaplasia. Pathology 2019;51:375–83. 31010590 10.1016/j.pathol.2019.02.001

[44] Saldías MP , Maureira D, Orellana-Serradell O, Silva I, Lavanderos B, Cruz P, . TRP channels interactome as a novel therapeutic target in breast cancer. Front Oncol 2021;11:621614. 34178620 10.3389/fonc.2021.621614 PMC8222984

[45] Emami Nejad A , Najafgholian S, Rostami A, Sistani A, Shojaeifar S, Esparvarinha M, . The role of hypoxia in the tumor microenvironment and development of cancer stem cell: a novel approach to developing treatment. Cancer Cell Int 2021;21:62. 33472628 10.1186/s12935-020-01719-5 PMC7816485

[46] Tutzauer J , Sjöström M, Holmberg E, Karlsson P, Killander F, Leeb-Lundberg LMF, . Breast cancer hypoxia in relation to prognosis and benefit from radiotherapy after breast-conserving surgery in a large, randomised trial with long-term follow-up. Br J Cancer 2021;126:1145–56. 10.1038/s41416-021-01630-4 PMC9023448 35140341

[47] Bassiouni R , Idowu MO, Gibbs LD, Robila V, Grizzard PJ, Webb MG, . Spatial transcriptomic analysis of a diverse patient cohort reveals a conserved architecture in triple-negative breast cancer. Cancer Res 2023;83:34–48. 36283023 10.1158/0008-5472.CAN-22-2682 PMC9812886

[48] Bandyopadhyay RS , Phelan M, Faller DV. Hypoxia induces AP-1-regulated genes and AP-1 transcription factor binding in human endothelial and other cell types. Biochim Biophys Acta 1995;1264:72–8. 7578260 10.1016/0167-4781(95)00116-x

[49] Zhang D , Iwabuchi S, Baba T, Hashimoto SI, Mukaida N, Sasaki SI. Involvement of a transcription factor, Nfe2, in breast cancer metastasis to bone. Cancers 2020;12:3003. 33081224 10.3390/cancers12103003 PMC7602858

[50] Archer MC . Role of sp transcription factors in the regulation of cancer cell metabolism. Genes Cancer 2011;2:712–9. 22207896 10.1177/1947601911423029 PMC3218407

[51] Pierre CC , Longo J, Bassey-Archibong BI, Hallett RM, Milosavljevic S, Beatty L, . Methylation-dependent regulation of hypoxia inducible factor-1 alpha gene expression by the transcription factor Kaiso. Biochim Biophys Acta 2015;1849:1432–41. 26514431 10.1016/j.bbagrm.2015.10.018

[52] Kantar D , Mur EB, Mancini M, Slaninova V, Salah YB, Costa L, . MAGI1 inhibits the AMOTL2/p38 stress pathway and prevents luminal breast tumorigenesis. Sci Rep 2021;11:5752. 33707576 10.1038/s41598-021-85056-1 PMC7952706

[53] Suzuki K , Tange M, Yamagishi R, Hanada H, Mukai S, Sato T, . SMG6 regulates DNA damage and cell survival in Hippo pathway kinase LATS2-inactivated malignant mesothelioma. Cell Death Discov 2022;8:446. 36335095 10.1038/s41420-022-01232-w PMC9637146

[54] Zhou Q , Lin H, Wang S, Wang S, Ran Y, Liu Y, . The ER-associated protein ZDHHC1 is a positive regulator of DNA virus-triggered, MITA/STING-dependent innate immune signaling. Cell Host Microbe 2014;16:450–61. 25299331 10.1016/j.chom.2014.09.006

[55] van Duzer A , Taniguchi S, Elhance A, Tsujikawa T, Oshimori N. ADAP1 promotes invasive squamous cell carcinoma progression and predicts patient survival. Life Sci Alliance 2019;2:e201900582. 31792062 10.26508/lsa.201900582 PMC6892435

[56] Bassey-Archibong BI , Hercules SM, Rayner LGA, Skeete DHA, Smith Connell SP, Brain I, . Kaiso is highly expressed in TNBC tissues of women of African ancestry compared to Caucasian women. Cancer Causes Control 2017;28:1295–1304. 28887687 10.1007/s10552-017-0955-2 PMC5681979

[57] Vermeulen JF , van de Ven RAH, Ercan C, van der Groep P, van der Wall E, Bult P, . Nuclear kaiso expression is associated with high grade and triple-negative invasive breast cancer. PLoS One 2012;7:e37864. 22662240 10.1371/journal.pone.0037864 PMC3360634

[58] Amith SR , Wilkinson JM, Fliegel L. Na+/H+ exchanger NHE1 regulation modulates metastatic potential and epithelial-mesenchymal transition of triple-negative breast cancer cells. Oncotarget 2016;7:21091–113. 27049728 10.18632/oncotarget.8520 PMC5008271

[59] Li Y , Zhou X, Liu J, Yin Y, Yuan X, Yang R, . Differentially expressed genes and key molecules of BRCA1/2-mutant breast cancer: Evidence from bioinformatics analyses. PeerJ 2020;8:e8403. 31998560 10.7717/peerj.8403 PMC6979404

[60] Liu L , Yan J, Cao Y, Yan Y, Shen X, Yu B, . Proliferation, migration and invasion of triple negative breast cancer cells are suppressed by berbamine via the PI3K/Akt/MDM2/p53 and PI3K/Akt/mTOR signaling pathways. Oncol Lett 2021;21:70. 33365081 10.3892/ol.2020.12331 PMC7716707

[61] Katayama A , Handa T, Komatsu K, Togo M, Horiguchi J, Nishiyama M, . Expression patterns of claudins in patients with triple-negative breast cancer are associated with nodal metastasis and worse outcome. Pathol Int 2017;67:404–13. 28699235 10.1111/pin.12560

[62] Batai K , Hooker S, Kittles RA. Leveraging genetic ancestry to study health disparities. Am J Phys Anthropol 2021;175:363–75. 32935870 10.1002/ajpa.24144 PMC8246846

[63] Davis M , Martini R, Newman L, Elemento O, White J, Verma A, . Identification of distinct heterogenic subtypes and molecular signatures associated with african ancestry in triple negative breast cancer using quantified genetic ancestry models in admixed race populations. Cancers 2020;12:1220. 32414099 10.3390/cancers12051220 PMC7281131

[64] Roelands J , Mall R, Almeer H, Thomas R, Mohamed MG, Bedri S, . Ancestry-associated transcriptomic profiles of breast cancer in patients of African, Arab, and European ancestry. NPJ Breast Cancer 2021;7:10. 33558495 10.1038/s41523-021-00215-x PMC7870839

