# Supplementary figures and images for: Chromatin Accessibility Landscape of Human Triple-negative Breast Cancer Cell Lines Reveals Variation by Patient Donor Ancestry
Source: Cancer Res Commun. 2023 Oct 5;3(10):2014–29. doi: 10.1158/2767-9764.CRC-23-0236 (PMC10552704; doi:10.1158/2767-9764.CRC-23-0236)

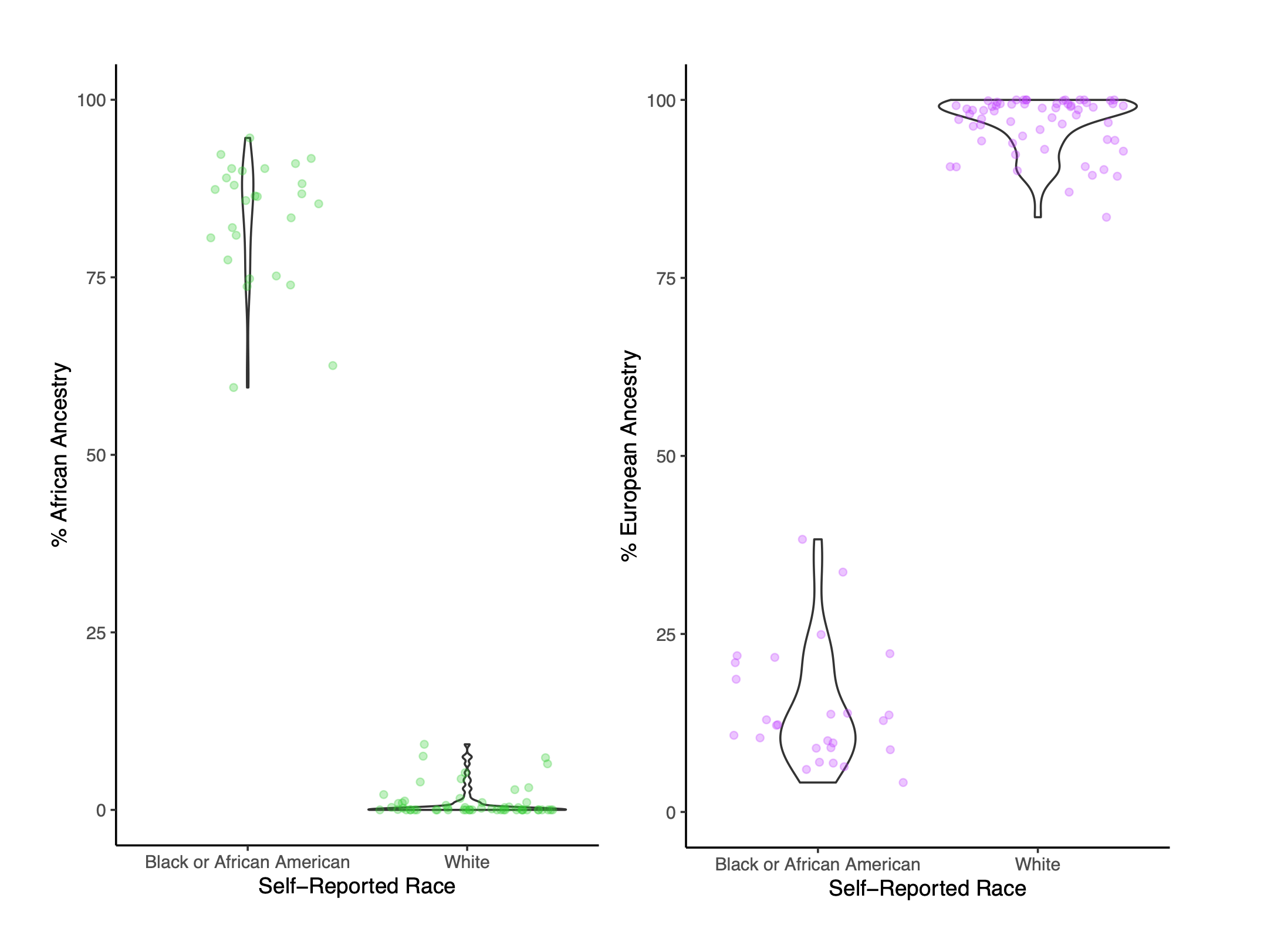

Supplement: Supplementary Figure 1 — Supplemental Figure 1. Quantified genetic ancestry in TCGA participants by self-reported race. Using published ancestry annotations from Carrot-Zhang et al1, quantified genetic ancestry was compared to self-reported race (SRR). For 111/112 TCGA samples used in our study, genetic ancestry consensus was concordant with SRR. Genetic ancestry consensus classification of African or admixed African ancestry captured 100% of TCGA participants who self-identified as “Black or African American”. Genetic ancestry consensus classification of EUR ancestry captured 98.7% of TCGA participants who self-identified as “White”; in one case, a participant that self-identified as White demonstrated a higher proportion of admixed American genetic ancestry. Among TCGA participants who self-identified as Black or African American, the average percentage of African ancestry was 83.2%, which is comparable to the quantified genetic ancestry estimates for the human cell lines used in this study that were classified as African American-derived (average African ancestry of 79.5%). Among TCGA participants who self-identified as White, the average percentage of European ancestry was 96.7%, which is comparable to the quantified genetic ancestry estimates for the human cell lines used in this study that were classified as European American-derived (average European ancestry of 93.0%). [file crc-23-0236-s01.png]

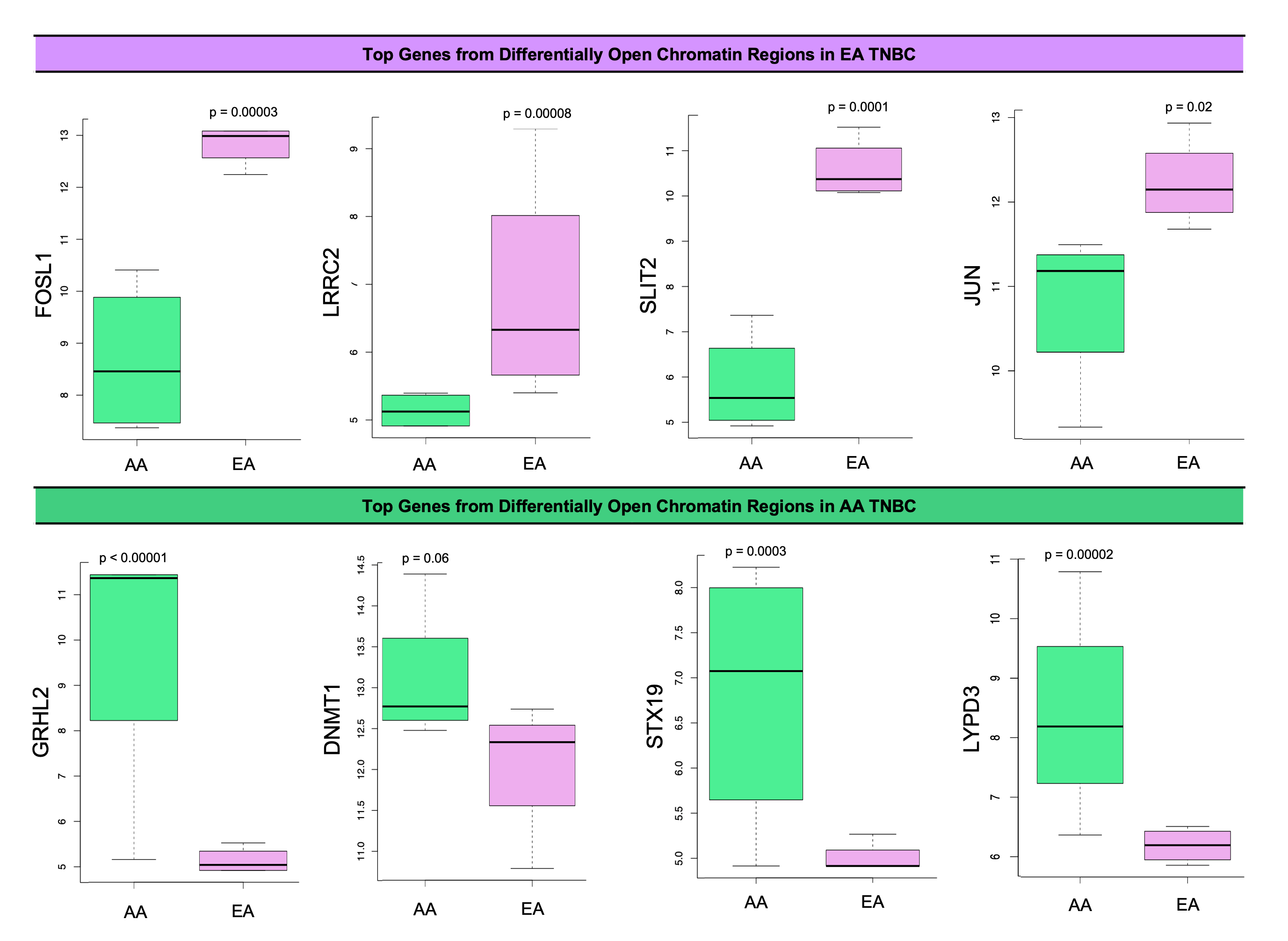

Supplement: Supplementary Figure 2 — Supplemental Figure 2. Expression of genes by donor ancestry in TNBC cell lines. Top-ranked genes identified to be located in open chromatin regions with an ancestry relationship were individually confirmed to be differentially expressed at the transcript level in matched RNA-seq data from the same human TNBC cell lines. Significance testing with two-sided t-test. [file crc-23-0236-s02.png]
